# Synergy of policies to strengthen primary care: evidence from a national repeated cross-sectional study

**DOI:** 10.1186/s12913-020-05695-4

**Published:** 2020-09-14

**Authors:** Yinzi Jin, Jin Xu, Weiming Zhu, Yaoguang Zhang, Ling Xu, Qingyue Meng

**Affiliations:** 1 School of Public Health, Peking University, 38 Xue Yuan Road, Haidian District, Beijing, 100191 China; 2 China Center for Health Development Studies, Peking University, Box 505, 38 Xue Yuan Road, Haidian District, Beijing, 100191 China; 3 Center for Health Statistics and Information, National Health Commission of the People’s Republic of China, 1 Hitch Man South Road, Xicheng District, Beijing, 100044 China

**Keywords:** Health insurance coverage, Health workforce availability, Synergic policies, Primary care strengthening

## Abstract

**Background:**

People bypass primary healthcare (PHC) institutions to seek expensive healthcare at high-level hospitals, leading to escalating medical costs and inefficient use of resources. In 2009, China launched nationwide synergic policies on primary care strengthening, to tackle access to healthcare and financial protection. This study aimed to assess the impact of the two policy areas, health insurance and health workforce, on healthcare seeking behavior.

**Methods:**

Drawing on national survey data before (2008) and after (2013) the policies, we linked individual-level data on healthcare-seeking behavior with county-level data on health workforce and health insurance. We constructed a multilevel zero-inflated negative binomial regression to examine the impacts of average reimbursement rate (ARR) of health insurance and the density of registered physicians on outpatient/inpatient visits, and multilevel multinomial logistic regression for the impacts on choice of outpatient/inpatient care providers.

**Results:**

Although the increase in health insurance ARR and physician density have positive impacts on individuals’ healthcare use, their impacts might be weakened during 2008 and 2013, and the negative impacts of investment of those in PHC institutions on likelihood of visiting hospitals was larger. The negative impacts of ARR at PHC institutions on likelihood of visiting county-, municipal- and higher-level hospitals in 2013 was 28 percentage points, 66 percentage points and 33 percentage points larger than these in 2008.

**Conclusions:**

Primary care strengthening requires synergic policies. Effective mechanisms for coordination across multisectoral actions are necessities for deepening those policies to ensure efficient delivery of healthcare without experiencing financial risks.

## Background

As Governments strive to progress towards the Sustainable Development Goals including Universal Health Coverage, concerted efforts are being made to strengthen primary healthcare (PHC) so that people have access to quality health services without experiencing financial risks. Synergy of policies on interrelated health systems function are warranted to strengthen PHC, especially for low- and middle-income countries (LMICs). Health financing and health workforce are two important policy areas. Healthcare seeking behavior refers to individuals’ use of health services to meet their health demands, and includes choosing from a range of services and optional healthcare providers [1]. Given that people desire good quality care at a low price, quality and price of health services are two important aspects for improving individuals’ healthcare seeking behaviors. Synergy of policies to strengthen PHC should focus on the quality and price of health services, guiding patients to choose appropriate healthcare providers for specific health services. Therefore, evaluating the changes of healthcare seeking behavior before and after the policies is of crucial importance to better understand the impact of the synergy of the policies—how health financing and health workforce promote the PHC strengthening—are worthy of thinking.

Before China’s health system reform in 2009, affected by the economic reforms since the 1970s, the health system had been once criticized for a massive reduction in financial health protection and substantial increases in out-of-pocket expenses. A large proportion of the population could not afford the healthcare they needed [2]. In 2009, China launched a nationwide comprehensive health system reform to improve affordable access to quality care [3]. Two major kinds of policies on primary care strengthening have been implemented. The first one is financing policy, related to the price of health services, with aims to expand healthcare coverage and the benefit package of the social health insurance schemes (SHI) for the population. SHI have set a gradient reimbursement rate where the rate at PHC institutions is higher than that at higher-level hospitals [4].

Another policy is focused on strengthening the availability of the PHC providers. Lack of qualified health workers is one of the causes for the poor quality of health services. In 2018, 25 and 42% of PHC providers in urban and rural areas, respectively, had less than a junior medical college level of education (the requirement for a licensed assistant physician) [5]. Therefore, enhancement of the availability of PHC physicians is essential for improving quality of PHC. The basic public health service program aims to deliver an essential public health services package to every Chinese citizen, in which governments subsidize the PHC providers based on the number of covered residents and the performance for service delivery. The national essential drug system aims to control the over-prescribing drugs, through eliminating mark-ups on drugs dispensed by PHC system. The local government has increased the budgets and introduced fixed salaries for PHC providers, to compensate for loss of income from drug sales [6]. Additionally, the local government has issued pay-for-performance scheme for PHC providers, a financial incentive which links part of income of PHC providers to the quality of their services, to attract qualified PHC providers [7, 8]. Chinese government has invested in health financing and health workforce in an integrated and systemic way, which are profoundly changing ways in which healthcare is financed and delivered [9].

The financing policy has made remarkable achievements. By the end of 2017, 95% of the population are covered by SHI, including New Rural Cooperative Medical Scheme (NRCMS), Urban Employee-based Medical Insurance (UEBMI), Urban Resident-based Medical Insurance (URBMI), and Urban and Rural Resident-based Medical Schemes (URRMS). Per capita fund for resident-based SHI increased from ¥100 in 2008 to ¥700 in 2018, about 70% from government subsides [10]. During the period of 2008 to 2017, people’s demand for health services was rapidly increased, with the outpatient and inpatient services utilization increased by 0.9 and 2.5 times, respectively [10]. But the proportion of outpatient and inpatient care provided by PHC institutions decreased by 9.6 and 10.0% points from 2008 to 2017, respectively [10]. Patients continue to bypass PHC system to seek expensive healthcare at high-level hospitals. As a result, although the proportion of out-of-pocket payments for healthcare decreased, the financial burden of using healthcare did not fall much, especially for poor households [11].

As two interrelated policy areas, health insurance coverage and health workforce availability have potential impacts on healthcare seeking behavior [12, 13]. The majority of studies in LMICs have focused on single dimension of health systems, for example, health financing mechanisms or strengthening of health workforce. Several studies focusing on strengthening PHC have qualitatively examined the impact of health systems approach for PHC delivery. However, to our knowledge, no studies have yet quantitively examined the impact of the synergy of policies (combined health system interventions) related to strengthening PHC on healthcare seeking behavior. Moreover, there is a value for data on policies at the county level because many health system reform policies are designed and implemented at this local level. To fill this research gap, this study aimed to estimate the impacts of the synergy of the policies, including health insurance coverage and health workforce, on individual’s health seeking behavior, and whether these impacts differed before and after the synergy of policies.

## Methods

### Data and sample

This study used two nationwide databases and linked individual-level data for the healthcare seeking behaviors, demographics, and socioeconomic characteristics with county-level data for health workforce and health insurance. Individual-level data were drawn from the China National Health Service Surveys (NHSS), which covered 94 counties with 177,501 respondents before (in 2008) and 156 counties with 273,687 respondents after (in 2013) the policies on primary care strengthening. The NHSS is a nationally representative survey that used four-stage stratified random cluster sampling. County-level data were reported by the health administrative departments of the counties sampled by the NHSS in 2008 and 2013. Both databases are managed by the National Health Commission (previously the Ministry of Health). In this study, individual- and county-level data were interconnected through administrative division codes.

### Measures and covariates

Healthcare seeking behaviors were measured by the number of outpatient/inpatient visits and the type of healthcare providers visited by outpatients/inpatients, including village/community health stations, township/community health centers, county hospitals, and municipal- or higher-level hospitals. PHC institutions refer to village clinics and township health centers in rural areas, and community health stations and community health centers in urban areas. In China, township/community health centers and hospitals offer inpatient services [14].

We measured health workforce density using the number of registered (assistant) doctors per 1000 population at county hospitals and PHC institutions. Health insurance coverage was measured by the SHI average reimbursement rate (ARR) of inpatient care at county hospitals and PHC institutions. ARRs were calculated using the mean of actual reimbursement rates for all inpatient services. We used ARR because inpatient reimbursement depends on patient copayment, the official reimbursement rate, and the ceiling. We also considered the variation of services and drug packages included in the SHI, which made the ARR for inpatient services more representative of the practical degree of SHI generosity.

Based on Andersen’s model [15–18] and empirical research [19–22], we controlled for variables that may act as potential confounders. We divided controlled factors into four components: predisposing factors, enabling factors, health needs, and environmental indicators. Predisposing factors included age (continuous variable), sex (male/female), marital status (single/married/divorced or widowed), education (no formal education/primary school/junior and high school/junior college and higher-level college), occupation (farmers/unemployed or retired/informal employed/formal employed). Enabling factors included income (continuous variable), health insurance status (NRCMS/UEBMI/URBMI/URRMS/none), distance to the nearest healthcare provider (less than 2 km/2–4 km/4- km and farther). Health needs included sickbed days for illness (continuous variable), presence of chronic diseases (yes/no). Environmental indicators included residence location (rural/urban).

### Statistical analysis

We compared the healthcare seeking behaviors, and the health insurance reimbursement and health workforce availability between 2008 and 2013. To account for unmeasured variations within counties, we applied multilevel random intercept analysis at individual level. Given the data type of the dependent variables and the number of outpatient visits with extra zeros and over-dispersion, multilevel zero-inflated negative binomial (ML_ZINB) regression was used to investigate the impact of health insurance and health workforce on outpatient/inpatient visits [23, 24]. Multilevel multinomial logistic (MML) regression was used to estimate the impact on outpatient/inpatient choice of healthcare providers, because the dependent variable was categorical random variable [25, 26]. In constructing the model, we assumed that individuals maximize their utilities through their decision-making processes [27]. For both of the regressions, two models were fitted: Model 1 regressed each outcome variable on health insurance, health workforce by year; Model 2 added additional regressors of the interaction terms between year and health insurance, year and health workforce. The sign of interaction term in Model 2 could be interpreted as whether the impacts differed before and after health system reform. Impact sizes as the result of the ML_ZINB regressions were presented as incidence rate ratio (IRR), whereas impact sizes of the MML regressions were expressed as relative risk ratio (RRR) [28]. All statistical analyses were performed using Stata 14.0.

## Results

### Healthcare seeking behaviors of study population

Table 1 showed the health needs and healthcare seeking behaviors before and after the synergic policies on primary care strengthening. For health needs, 18.9 and 24.1% of adults reported a sick within 2 weeks, and 24.1 and 33.1% had any chronic disease, respectively in 2008 and 2013. Among those in a need for outpatient care, 39.1% took outpatient care, 27.1% took self-medication, 23.3% continued treatment that took two weeks, and 10.6% took no treatment in 2008; while the proportions of those were 37.2, 14.1, 47.2 and 1.4% in 2013. The admissions within 1 year rose from 6.8% in 2008 to 9.0% in 2013. The proportion of patients choosing healthcare institutions within the county (PHC institutions and county hospitals) decreased from 76.9% in 2008 to 72.6% in 2013. Characteristics of samples were shown in **Appendix Table 1.**

**Table 1:** Health needs and healthcare seeking behaviors before and after China’s health system reform

	2008	2013	Relative change (%)	*P* value
**Health needs**				
Prevalence within 2 weeks (mean‰[mean ± SD])	18.9 (15.1, 22.8)	24.1 (19.9, 28.3)	27.5%	< 0.001
Prevalence of chronic diseases (mean‰[mean ± SD])	24.1 (20.4, 27.8)	33.1 (29.0, 37.2)	37.3%	< 0.001
**Outpatient healthcare seeking behavior**				
Outpatient visits within 2 weeks (mean%[mean ± SD])	14.5 (8.4, 20.6)	13.0 (7.9, 18.1)	−10.3%	0.105
healthcare-seeking behaviors for people in a need for outpatient care (%)				< 0.001
Outpatient care	39.1	37.2	−4.8%	
Self-medication	27.1	14.1	−48.0%	
Continued treatment that took two weeks before	23.3	47.2	102.6%	
No treatment	10.6	1.4	− 86.8%	
The percentage of non-treatment or self-medication due to financial difficulties (%)	24.4	13.6	−44.3%	< 0.001
Percentage of outpatient healthcare provider (%)				< 0.001
Village/community healthcare stations	49.5	50.2	1.4%	
Township/community healthcare centers	24.2	22.4	−7.4%	
County hospitals	17.3	16.9	−2.3%	
Municipal and higher-level hospitals	8.9	10.5	18.0%	
**Inpatient healthcare seeking behavior**				
Admissions within 1 year (mean%[mean ± SD])	6.8 (6.5–7.1)	9.0 (8.6–9.4)	32.4%	< 0.001
The percentage of people reported a need for admission but did not receive inpatient care (%)	25.1	17.1	− 31.9%	< 0.001
The percentage of non-hospitalized due to financial difficulties (%)	70.3	43.2	−38.5%	< 0.001
Percentage of inpatient healthcare provider (%)				< 0.001
Township/community healthcare centers	28.7	21.0	−26.8%	
County hospitals	48.2	51.6	7.1%	
Municipal hospitals	11.9	17.9	50.4%	
Provincial hospitals	8.2	7.3	−11.0%	
N	177,501	273,687	–	

### Health insurance reimbursement and health workforce availability

**Fig. 1:**
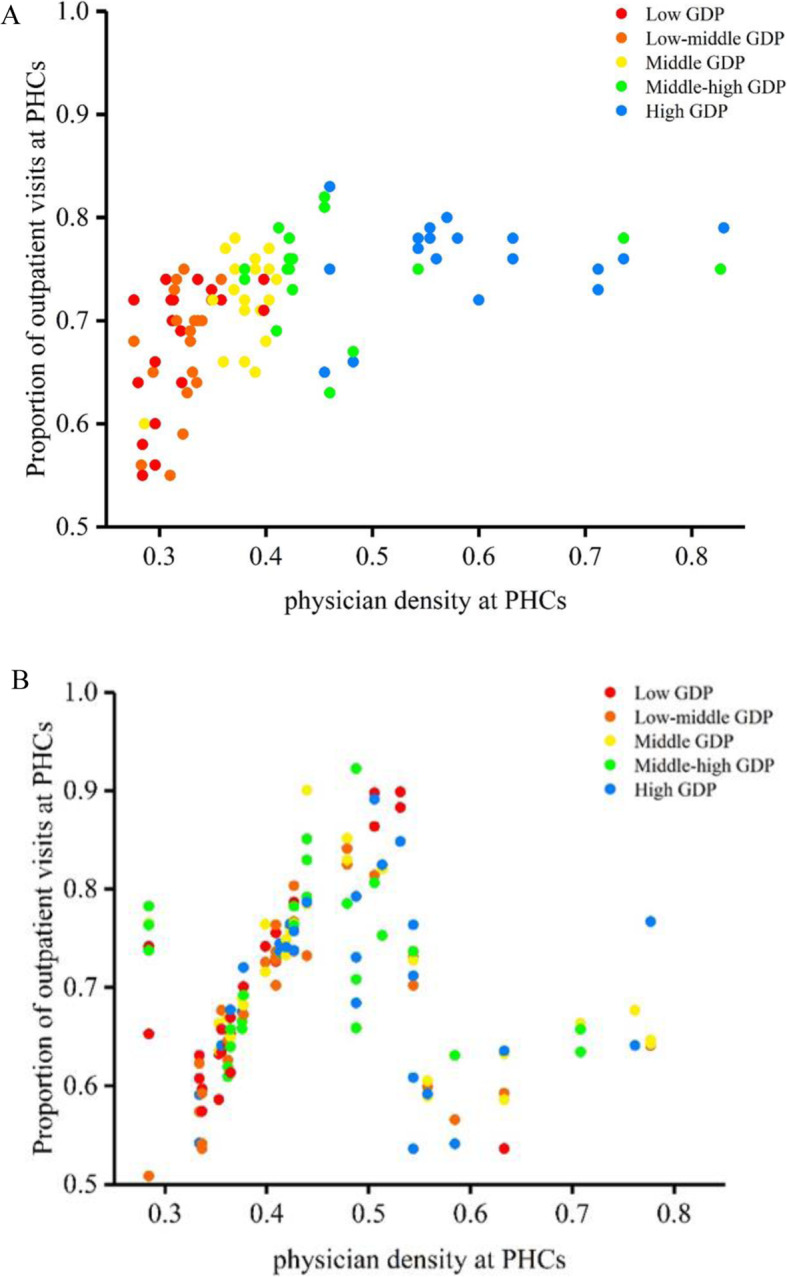
Comparing the proportion of outpatient visits at PHC institutions to physician density at PHC institutions by GDP group, 2008 (**A**) and 2013 (**B**)

From 2008 to 2013, the health insurance ARR increased from 48 to 64%. The ARRs at county hospitals (40% in 2008 and 52% in 2013) were lower than these at higher level hospitals (45% in 2008 and 57% in 2013). The physician density in municipal- and higher-level hospitals increased by 1.21 compared with 0.73 in county hospitals and 0.03 at PHC institutions, revealing a widening gap between PHC institutions and hospitals from 2008 to 2013 (Table 2). Counties with low physician density at PHC institutions had lower proportion of outpatient visits at PHC institutions than those with high physician density both in 2008 and 2013 (Fig. 1).

**Table 2:** Health insurance and health workforce before and after China’s health system reform

		2008			2013	
	Urban	Rural	Total	Urban	Rural	Total
Health insurance						
health insurance coverage (%)	66.20	91.90	87.10	91.60	97.30	95.20
ARR at PHC institutions, Mean ± SD	0.52 (0.34, 0.71)	0.47 (0.25, 0.69)	0.48 (0.26, 0.71)	0.64 (0.41, 0.86)	0.66 (0.44, 0.89)	0.64 (0.42, 0.86)
ARR at county hospitals, Mean ± SD	0.45 (0.34, 0.56)	0.35 (0.24, 0.46)	0.40 (0.27, 0.53)	0.58 (0.49, 0.67)	0.48 (0.40, 0.55)	0.52 (0.44, 0.60)
ARR at higher level hospitals, Mean ± SD	0.45 (0.27, 0.63)	–	0.45 (0.27, 0.63)	0.57 (0.41, 0.74)	–	0.57 (0.41, 0.75)
Health workforce						
Physicians density at PHCs, Mean ± SD	0.46 (0.42–0.50)	0.39 (0.35–0.43)	0.41 (0.36–0.46)	0.56 (0.51–0.61)	0.37 (0.34–0.40)	0.44 (0.40–0.48)
Physicians density at county hospitals, Mean ± SD	1.96 (1.89–2.03)	0.55 (0.51–0.59)	0.74 (0.68–0.80)	2.62 (2.49–2.75)	0.80 (0.76–0.84)	1.47 (1.43–1.51)
Physicians density at higher level hospitals, Mean ± SD	3.68 (2.56, 4.80)	–	3.68 (2.56, 4.80)	4.89 (3.25, 6.53)	–	4.89 (3.25, 6.53)
N	35	59	94	78	78	156

### Impact of health insurance and health workforce before and after the synergic policies

Table 3 shows the results of ML_ZINB regressions. After adjusting for potential confounders, a higher ARR at PHC institutions, physician density at PHC institutions and physician density at county hospitals were associated with a 19% (IRR = 1.19, 95% CI = 1.12–1.27, *p* < 0.001), 13% (IRR = 1.13, 95% CI = 1.04–1.22, p < 0.001) and 38% (IRR = 1.38, 95% CI = 1.29–1.48, p < 0.001) higher outpatient visits in 2008; while in 2013, the IRRs were 1.04 (95%CI = 0.99–1.10, p < 0.001), 1.11 (95%CI = 1.05–1.18, *p* < 0.001) and 1.08 (95%CI = 1.02–1.15, p < 0.001), respectively. The rising health insurance ARR and physician density at PHC institutions were positively and significantly associated with healthcare use. After adding the interaction terms with year, the IRR of interaction terms were lower than 1 (p < 0.001). In other words, compared to 2008, the positive impacts of ARR and physician density on increasing outpatient and inpatient visits dropped significantly in 2013, after controlling for other covariates (**Appendix Table 2**). **Appendix Table 4** shows the results of regressions before and after China’s health system reform, respectively.

**Table 3:** Impact of health insurance and health workforce on healthcare use before and after China’s health system reform

	Self-medication (IRR)		Outpatient visits (IRR)		Hospital admissions (IRR)	
	Before China’s health system reform	After China’s health system reform	Before China’s health system reform	After China’s health system reform	Before China’s health system reform	After China’s health system reform
ARR at PHC institutions	0.99* (0.99, 1.00)	0.91** (0.90, 0.93)	1.19*** (1.12, 1.27)	1.04 (0.99, 1.10)	1.55*** (1.28, 1.88)	1.42 *** (1.27, 1.59)
ARR at county hospitals	0.96*** (0.94, 0.98)	0.90*** (0.88, 0.92)	1.16*** (1.08, 1.23)	1.01 (0.96, 1.06)	1.82*** (1.47, 2.25)	1.02 (0.77, 1.36)
Physicians density at PHC institutions	0.79* (0.77, 0.81)	0.83** (0.81, 0.85)	1.13*** (1.04, 1.22)	1.11*** (1.05, 1.18)	1.61 *** (1.41, 1.84)	1.00 (0.98, 1.01)
Physicians density at county hospitals	0.99 (0.92, 1.06)	0.91* (0.88, 0.95)	1.38*** (1.29, 1.48)	1.08*** (1.02, 1.15)	1.03 ** (1.00, 1.05)	0.98 ** (0.97, 0.99)

*p < 0.05, **p < 0.01, ***p < 0.001

Table 4 shows the RRRs for MML regressions. The rising health insurance ARR and physician density at PHC institutions were negatively and significantly associated with the probability of visiting county and higher-level hospitals. When the ARR at PHC institutions increased by 1 percentage point, the likelihood of inpatients choosing county-, municipal- and higher-level hospitals decreased by 25% (RRR = 0.75, 95% CI = 0.57–0.99, *p* < 0.001), 45% (RRR = 0.55, 95% CI = 0.37–0.82, p < 0.001) and 46% (RRR = 0.54, 95% CI = 0.36–0.82, p < 0.001), respectively, in 2008; while in 2013, the RRRs were 0.54 (95%CI = 0.44–0.66), 0.19 (95%CI = 0.15–0.24), and 0.37 (95%CI = 0.27–0.50), respectively. The results of interaction terms indicated that the negative impact of ARR at PHC institutions on likelihood of visiting county-, municipal- and higher-level hospitals in 2013 was 28, 66 and 33% larger than these in 2008. Similar patterns were also observed for physician density at PHC institutions and county hospitals (**Appendix Table 3**). **Appendix Table 5** shows the results of regressions among outpatient care in 2008 and 2013, respectively, and **Appendix Table 6** shows the results of regressions among inpatient care.

**Table 4:** Impact of health insurance and health workforce on choice of healthcare providers before and after China’s health system reform

Outpatient choice of healthcare providers (reference group is village/community health stations)	Before China’s health system reform (RRR)			After China’s health system reform (RRR)		
	Township/ community health centers	County hospitals	Municipal or higher-level hospitals	Township/ community health centers	County hospitals	Municipal or higher-level hospitals
ARR at PHC institutions	1.44 (0.96, 2.16)	0.28*** (0.18, 0.44)	0.18*** (0.10, 0.31)	1.76 *** (1.04, 2.74)	1.07 (0.80, 1.44)	0.98 (0.73, 1.32)
ARR at county hospitals	1.31 (0.71, 2.39)	1.68 (0.90, 3.15)	2.92*** (1.51, 5.66)	1.44* (1.00, 2.08)	1.72*** (1.20, 2.45)	1.87*** (1.27, 2.74)
Physicians density at PHC institutions	1.23*** (1.15, 1.32)	0.43*** (0.30, 0.61)	0.76 (0.43, 1.33)	1.34 (0.80, 2.24)	0.95*** (0.91, 0.99)	0.98 (0.95, 1.02)
Physicians density at county hospitals	1.02 (0.98, 1.06)	1.38*** (1.29, 1.48)	1.45 *** (1.33, 1.58)	0.91*** (0.89, 0.94)	0.98 (0.95, 1.01)	0.99 (0.96, 1.02)
**Inpatient choice of healthcare providers (reference group is township/community healthcare centers)**	**County** **hospitals**	**Municipal hospitals**	**Provincial or higher-level hospitals**	**County** **hospitals**	**Municipal hospitals**	**Provincial or higher-level hospitals**
ARR at PHC institutions	0.75** (0.57, 0.99)	0.55*** (0.37, 0.82)	0.54*** (0.36, 0.82)	0.54*** (0.44, 0.66)	0.19*** (0.15, 0.24)	0.37*** (0.27, 0.50)
ARR at county hospitals	1.22*** (1.06, 1.41)	1.76*** (1.27, 2.45)	1.85*** (1.34, 2.56)	1.12 (0.91, 1.38)	1.68*** (1.39, 2.03)	2.18*** (1.74, 2.73)
Physicians density at PHC institutions	0.90*** (0.84, 0.97)	0.84*** (0.78, 0.91)	0.92* (0.85, 1.00)	0.27*** (0.19, 0.36)	0.18*** (0.10, 0.30)	0.28*** (0.16, 0.48)
Physicians density at county hospitals	1.41*** (1.29, 1.53)	1.48*** (1.34, 1.64)	1.52*** (1.37, 1.69)	1.12*** (1.07, 1.17)	1.14*** (1.09, 1.20)	1.16*** (1.11, 1.22)

*p < 0.05, **p < 0.01, ***p < 0.001

## Discussion

To the best of our knowledge, this is the first study evaluating the impact of health policies on healthcare seeking behaviors, from the perspective of synergic policies to strengthening primary care [29]. Taking advantage of the population-based nationally representative survey before and after 2009, we were able to investigate whether the impacts changed with the progress of the health system reform, in which the two policy areas are evolving simultaneously. Our findings can provide implications for further advancing the agenda of deepening the synergic policies on primary care strengthening, by identifying policy entry points to promote PHC systems from a joint vision.

In this study, several key findings were highlighted. First, increasing health insurance ARR and physician density at PHC institutions were associated with more outpatient visits and admissions and more likelihood of visiting PHC institutions. Previous studies have found that introduction of PHC providers would lead to a shift of care from specialists to primary care for disease management in different settings [30–34]. In addition, studies in Sweden and Mexico have indicated that government financial investment in PHCs increased the number of visits to PHCs [12, 35]. The majority of studies have focused on single policies (e.g. health insurance or investment in health workforce). However, few have investigated the efforts in a synergic way which contributed to improving use of PHC of a large proportion of the world’s population [36]. Strengthening primary care requires national actions in multiple interrelated health systems policy areas [37]. Increasing the use of PHC may be dependent on improvements in service delivery, including the management of health workforce, as well as in financing. Recognizing these interdependencies makes the task of designing or reforming systems a complex one, but is critical for a systemic approach to primary care strengthening.

Second, compared to 2008, the positive impacts of health insurance ARR and physician density on increasing outpatient visits and admissions dropped significantly in 2013. Diminished marginal return of increasing health inputs in high-resource-density domains implies better strategies that priorities of health resource allocation need to focus on the resource-poor parts such as the PHC institutions [38]. The findings on comparison in healthcare seeking behaviors between 2008 and 2013 were consistent with previous studies focusing on China’s health system reform, which indicated that the reform with multipartite policies may make interactional impacts on healthcare use [39]. Nevertheless, these previous studies have not assessed the impact of health system reform on healthcare seeking behaviors. Notably, the positive associations with admissions still reminded us with the challenge that a moral hazard situation arises when health insurance coverage is universal, and SHI participants overuse health services especially hospital-based medical services [40–42]. In 2016, the rate of hospital admission in China was 16.4%, higher than the average for countries of the Organization for Economic Cooperation and Development, which implies that China needs to examine the appropriateness of inpatient care, including overuse and misuse of medical services. Therefore, China’s health system reform needs to consider redistributing the existing health resources rather than to continuously increase the health resources, for more effectiveness of financial and service-delivery policy arrangements. A previous study showed the encouraging results that the investment in PHC providers showed largest impact on improving healthcare use [43], so ensuring an adequate availability of PHC providers is one of top priorities to improve the effectiveness of healthcare delivery. Our study provided an evidence-based approach for taking steps towards structural adjustment to tackle the sluggish development of existing policy arrangements [44].

Third, the negative impact of health insurance ARR and physician density at PHC institutions on likelihood of visiting hospitals in 2013 was larger than these in 2008. The results can be explained by the declining use of PHC as a proportion of total health services from 2008 to 2013. Despite the evidence of the progress made in strengthening the PHC system, some challenges remain immense. The physician density at county hospitals was higher than PHC institutions, and had also seen a higher growth, thereby widening the gap of physician density between PHC institutions and hospitals from 2008 to 2013. This unintended result of the reform might lead to the declining use of PHC as a proportion of total health services. The major reason for the unintended results of the reform is the inconsistence of the development of health insurance and health workforce, the two health system policy areas. Although the SHI has achieved a lot in coverage and service benefit, the quantity of PHC providers is inadequate [9, 45], and the incentive mechanism for PHC providers is weak [8].

There are three points on the implementation of policies to explain how the real condition, unlike the policy, are related to the findings of the study. First is the zero-profit medicine policy [6]. Although local governments increase the budgets to balance financial loss of PHC institutions from drugs benefits, this part of financial support is dependent on local government’s financial capacity and cannot make up the loss in most of cases [46]. There are many complaints about the unavailability of essential drugs on the list of the SHI, that pushes patients to be referred to hospitals and restricts the professional development of PHC providers [47]. Although the SHI has achieved a lot in coverage and service benefit, the limitation of essential drugs covered by the SHI can lead patients bypass PHC institutions to seek health services at high-level hospitals. Second is the financial arrangements for PHC institutions with the delink between revenue and expenditure. The revenues obtained by providing PHC should be turned over to the government financial accounts, and the expenditures incurred are financed according to the standards designed by the government financial department [7]. The delink between revenue and expenditure reduced the financial incentives for PHC providers because their income is fixed and has nothing connection with the workload of providing PHC [46]. However, the fee-for-service payment system in hospitals gives hospitals an incentive to attract and retain patients who could otherwise use PHC providers [9]. Third is the salary reform for PHC providers. The percentage of performance-based bonus on the total income is quite low, limiting the financial incentives for providing PHC [8]. The consequent lack of motivation has led to a brain drain to hospitals and out of the health system altogether [48]. In 2017, only 13% of PHC providers had a formal medical education (five years of medical school) in rural and 40% in urban areas [10]. In a word, efforts to cope with the capacity strengthening PHC system have been slow, mostly because of insufficient coordination and fragmented systems [4, 49].

Since quality of care given by PHC providers is still unsatisfactory, patients in real needs choose to bypass the PHC system in favor of hospitals, which resulted in soaring cost of medical care [50]. The synergic policies that are issued to tackle access to healthcare and financial protection have not succeed, even further lower the affordable accessibility of the low-income group. Therefore, further reforms should consider transforming the existing hospital-centered healthcare system to an integrated health system based on PHC in a systemic way. A competent health workforce is indisputably important, and a good financing system including effective incentive mechanisms for PHC providers should continue to focus on aligning incentives for providing quality PHC [51]. Strengthening platforms to design and implement more effective multisectoral actions is urgently required. This can include the development of national whole-of-government multisectoral plans, establishing mechanisms for coordination across ministries and other stakeholders, and multi-sectoral mechanism at the stage of monitoring and evaluating enforcement of policies [52].

This study has several limitations. First, the observational nature of our study limited our ability to draw any causal inference from our findings. Rather, the association found in this study underscored the need for research to evaluate the progress of the synergic policies on primary care strengthening from the perspectives of health financing and health workforce. Second, only the 2008 and 2013 round of NHSS were included to evaluate the five-year progress of health system policies. This mid-term impact assessment might limit us to generate policy relevance. Although we did not have data of the latest 2018 round of NHSS which has not been open for analysis, it was reported that healthcare seeking behavior sustained the trend and the health insurance coverage and physician density continued to be improved during 2013 and 2018 [9, 11]. Nonetheless, this interim impact analysis might make our estimates of the associations between health insurance and health workforce and healthcare seeking behavior conservative.

## Conclusions

Primary care strengthening requires synergic policies. Our findings highlighted the role of strengthening PHC on improving the effectiveness of financial and service-delivery policy arrangements, and the declining use of PHC as a proportion of total health services could be attributable to the inconsistent development of the two policy areas. Effective mechanisms for coordination across multisectoral actions in an integrated and systemic way are required for deepening those policies to ensure efficient delivery of high-quality healthcare without experiencing financial risks. The implications can guide decision-making on the entry points to reinforce PHC planning, resource allocation, and service delivery in various LMICs.

## Supplementary information

**Additional file 1 Appendix Table 1** Sample characteristics. **Appendix Table 2** Impact of health insurance and health workforce on healthcare use with interaction terms of year before and after China’s health system reform. **Appendix Table 3** Impact of health insurance and health workforce on choice of healthcare providers with interaction terms of year before and after China’s health system reform. **Appendix Table 4** Impact of health insurance and health workforce on healthcare use before and after China’s health system reform. **Appendix Table 5** Impact of health insurance and health workforce on choice of healthcare providers among outpatient care before and after China’s health system reform. **Appendix Table 6** Impact of health insurance and health workforce on choice of healthcare providers among inpatient care before and after China’s health system reform

## Data Availability

The datasets are not publicly available but are available from the National Health Commission on reasonable request.

## Abbreviations

LMICs: low- and middle-income countries
SHI: social health insurance schemes
PHC: primary healthcare
NHSS: National Health Service Surveys
ARR: average reimbursement rate
ML_ZINB: multilevel zero-inflated negative binomial
MML: multilevel multinomial logistic
IRR: incidence rate ratio
RRR: relative risk ratio
