# Supplementary material for: Synergy of policies to strengthen primary care: evidence from a national repeated cross-sectional study
Source: BMC Health Serv Res. 2020 Sep 14;20:865. doi: 10.1186/s12913-020-05695-4 (PMC7488991; doi:10.1186/s12913-020-05695-4)
Supplement: Supplementary file 1 — Additional file 1 Appendix Table 1 Sample characteristics. Appendix Table 2 Impact of health insurance and health workforce on healthcare use with interaction terms of year before and after China’s health system reform. Appendix Table 3 Impact of health insurance and health workforce on choice of healthcare providers with interaction terms of year before and after China’s health system reform. Appendix Table 4 Impact of health insurance and health workforce on healthcare use before and after China’s health system reform. Appendix Table 5 Impact of health insurance and health workforce on choice of healthcare providers among outpatient care before and after China’s health system reform. Appendix Table 6 Impact of health insurance and health workforce on choice of healthcare providers among inpatient care before and after China’s health system reform [file 12913_2020_5695_MOESM1_ESM.docx]

**Appendix Table 1** Sample characteristics

|  |  | 2008 |  |  |  | 2013 |  |
| --- | --- | --- | --- | --- | --- | --- | --- |
|  | Urban | Rural | Total |  | Urban | Rural | Total |
| Rural (%) | -- | -- | 73.8 |  | -- | -- | 51.3 |
| Female (%) | 48.6 | 55.6 | 50.0 |  | 51.2 | 49.8 | 50.5 |
| Age (%) |  |  |  |  |  |  |  |
| -14 | 11.9 | 19.6 | 17.7 |  | 13.7 | 18.0 | 16.0 |
| 15-44 | 40.3 | 43.1 | 42.3 |  | 37.1 | 34.9 | 36.0 |
| 45-64 | 33.2 | 34.0 | 28.5 |  | 31.6 | 27.5 | 33.6 |
| 65- | 16.3 | 9.8 | 11.5 |  | 16.1 | 13.1 | 14.5 |
| Currently Married (%) | 64.4 | 60.8 | 61.7 |  | 68.6 | 64.7 | 66.8 |
| Education (%) |  |  |  |  |  |  |  |
| No formal education | 6.1 | 14.8 | 12.9 |  | 4.3 | 14.0 | 10.3 |
| Primary school | 24.8 | 44.8 | 39.3 |  | 23.1 | 44.9 | 36.1 |
| Junior and high school | 61.4 | 39.9 | 42.2 |  | 62.5 | 40.4 | 44.5 |
| Junior college and above | 7.7 | 0.7 | 5.4 |  | 10.1 | 0.6 | 9.2 |
| Employment status (%) |  |  |  |  |  |  |  |
| Farmers | 3.7 | 51.1 | 46.9 |  | 3.2 | 52.4 | 45.7 |
| Unemployed | 25.8 | 15.1 | 21.2 |  | 27.1 | 16.7 | 22.1 |
| Informal employed | 23.7 | 9.1 | 19.5 |  | 30.3 | 12.0 | 17.3 |
| Formal employed | 41.8 | 5.6 | 12.4 |  | 45.5 | 6.8 | 14.9 |
| Household income per capita (1000RMB) ^a^ | 11.0 (1.8) | 5.2 (1.2) | 6.6 (1.5) |  | 20.3 (3.7) | 10.2 (2.7) | 14.1 (3.2) |
| Health insurance status (%) |  |  |  |  |  |  |  |
| NCMS | 11.8 | 89.5 | 68.7 |  | 5.2 | 79.1 | 51.1 |
| URRMS | -- | -- | -- |  | 5.0 | 14.9 | 9.9 |
| URBMI | 10.6 | 0.7 | 3.8 |  | 28.1 | 2.7 | 13.2 |
| UEBMI | 46.3 | 1.8 | 13.7 |  | 54.2 | 1.7 | 21.0 |
| None | 31.3 | 8.0 | 12.9 |  | 7.5 | 1.6 | 4.4 |
| N | 46,510 | 118,991 | 177,501 |  | 133,393 | 140,295 | 273,687 |

NCMS, New Rural Cooperative Medical Schemes; URRMS, Urban and Rural Resident-based Medical Schemes; URBMI, Urban Resident-based Basic Medical Insurance; UEBMI, Urban Employee-based Basic Medical Insurance.

^a^ Mean, standard deviation (SD)

**Appendix Table 2** Impact of health insurance and health workforce on healthcare use with interaction terms of year before and after China’s health system reform

|  | Outpatients visits (IRR) | |  | Hospital admissions (IRR) | | |
| --- | --- | --- | --- | --- | --- | --- |
|  | Model without interaction | Model with interaction |  | Model without interaction | Model with interaction |  |
| ARR at PHC institutions | 0.87 ***  (0.86, 0.88) | 1.33 ***  (1.20, 1.50) |  | 1.37 ***  (1.25, 1.51) | 1.41 ***  (1.25, 1.68) |  |
| ARR at county hospitals | 0.87 ***  (0.86, 0.89) | 1.12 ***  (1.07, 1.17) |  | 1.30 ***  (1.11, 1.54) | 1.34 ***  (1.19, 1.52) |  |
| Physicians density at PHC institutions | 0.98 ***  (0.97, 1.01) | 1.09 *  (0.99, 1.24) |  | 1.00  (0.99, 1.02) | 1.01**  (1.00, 1.02) |  |
| Physicians density at county hospitals | 0.66 ***  (0.65, 0.66) | 1.00  (0.98, 1.02) |  | 0.98  (0.96, 1.01) | 1.01  (0.99, 1.03) |  |
| ARR at PHC institutions × year |  | 0.97 ***  (0.95, 0.98) |  |  | 0.92 ***  (0.84, 0.99) |  |
| ARR at county hospitals × year |  | 0.87 ***  (0.86, 0.88) |  |  | 0.78 ***  (0.65, 0.91) |  |
| Physicians density at PHC institutions × year |  | 0.86 ***  (0.85, 0.88) |  |  | 0.62 ***  (0.55, 0.72) |  |
| Physicians density at county hospitals × year |  | 0.81***  (0.79, 0.82) |  |  | 0.95 ***  (0.94, 0.97) |  |

**Appendix Table 3** Impact of health insurance and health workforce on choice of healthcare providers with interaction terms of year before and after China’s health system reform

|  | Healthcare providers for outpatient care (RRR) | | |  | Healthcare providers for inpatient care (RRR) | | |
| --- | --- | --- | --- | --- | --- | --- | --- |
|  | Township/  community healthcare centers | County hospitals | Municipal hospitals |  | County hospitals | Municipal hospitals | Provincial hospitals |
| Model without interaction |  |  |  |  |  |  |  |
| ARR at PHC institutions | 2.37***  (1.87, 3.00) | 1.34**  (1.03, 1.76) | 1.02  (0.98, 1.06) |  | 0.65 ***  (0.55, 0.76) | 0.27 ***  (0.22, 0.32) | 0.45 ***  (0.36, 0.57) |
| ARR at county hospitals | 1.02  (0.95, 1.08) | 1.54***  (1.14, 2.08) | 1.97***  (1.43, 2.71) |  | 1.23 ***  (1.10, 1.38) | 1.72 ***  (1.46, 2.02) | 2.14 ***  (1.78, 2.57) |
| Physicians density at PHC institutions | 1.53***  (1.21, 1.93) | 0.92***  (0.88, 0.96) | 0.94***  (0.91, 0.98) |  | 0.85 ***  (0.80, 0.90) | 0.78 ***  (0.73, 0.84) | 0.86 ***  (0.80, 0.92) |
| Physicians density at county hospitals | 0.94***  (0.91, 0.96) | 1.01  (0.99, 1.04) | 1.03**  (1.00, 1.06) |  | 1.17 ***  (1.13, 1.22) | 1.21 ***  (1.16, 1.26) | 1.23 ***  (1.17, 1.28) |
| Model with interaction |  |  |  |  |  |  |  |
| ARR at PHC institutions | 1.10***  (1.03, 1.16) | 0.63***  (0.61, 0.67) | 0.48***  (0.43, 0.53) |  | 0.96 ***  (0.92, 1.00) | 0.90 ***  (0.87, 0.91) | 0.84 ***  (0.80, 0.89) |
| ARR at county hospitals | 0.97  (0.89, 1.04) | 1.20***  (1.18, 1.21) | 1.63***  (1.61, 1.66) |  | 1.13 ***  (1.03, 1.27) | 1.29 ***  (1.28, 1.30) | 1.30 ***  (1.25, 1.35) |
| Physicians density at PHC institutions | 1.16*  (1.10, 1.35) | 0.68***  (0.61, 0.77) | 0.87  (0.70, 1.08) |  | 0.98 ***  (0.97, 0.99) | 1.10  (0.95, 1.16) | 0.98  (0.84, 1.01) |
| Physicians density at county hospitals | 1.02**  (1.00, 1.04) | 1.17***  (1.16, 1.18) | 1.20***  (1.18, 1.21) |  | 1.19 ****  (1.18, 1.20) | 1.22 ***  (1.21, 1.23) | 1.23 ***  (1.22, 1.24) |
| ARR at PHC institutions × year | 1.92***  (1.73, 2.12) | 3.82***  (3.27, 4.44) | 5.44***  (4.26, 7.30) |  | 0.72 ***  (0.67, 0.76) | 0.34 ***  (0.30, 0.40) | 0.67 ***  (0.61, 0.74) |
| ARR at county hospitals × year | 1.10  (0.87, 1.41) | 1.02  (0.78, 1.33) | 0.64***  (0.48, 0.84) |  | 0.92  (0.64, 1.30) | 0.95  (0.83, 1.10) | 1.18 ***  (1.07, 1.29) |
| Physicians density at PHC institutions × year | 1.08  (0.61, 1.95) | 2.21***  (1.62, 3.03) | 1.29  (0.77, 2.21) |  | 0.90 ***  (0.86, 0.96) | 0.62 ***  (0.52, 0.94) | 0.90 ***  (0.78, 1.38) |
| Physicians density at county hospitals × year | 0.89***  (0.84, 0.96) | 0.71***  (0.68, 0.74) | 0.68***  (0.64, 0.72) |  | 0.79 ***  (0.76, 0.83) | 0.77 ***  (0.73, 0.82) | 0.77 ***  (0.72, 0.81) |

**Appendix Table 4** Impact of health insurance and health workforce on healthcare use before and after China’s health system reform

|  | Self-medication (IRR) | |  | Outpatient visits (IRR) | |  | Hospital admissions (IRR) | |
| --- | --- | --- | --- | --- | --- | --- | --- | --- |
|  | Before China’s health system reform | After China’s health system reform |  | Before China’s health system reform | After China’s health system reform |  | Before China’s health system reform | After China’s health system reform |
| ARR at PHC institutions | 0.99*  (0.99, 1.00) | 0.91**  (0.90, 0.93) |  | 1.19***  (1.12, 1.27) | 1.04  (0.99, 1.10) |  | 1.55***  (1.28, 1.88) | 1.42 ***  (1.27, 1.59) |
| ARR at county hospitals | 0.96***  (0.94, 0.98) | 0.90***  (0.88, 0.92) |  | 1.16***  (1.08, 1.23) | 1.01  (0.96, 1.06) |  | 1.82***  (1.47, 2.25) | 1.02  (0.77, 1.36) |
| Physicians density at PHC institutions | 0.79*  (0.77, 0.81) | 0.83**  (0.81, 0.85) |  | 1.13***  (1.04, 1.22) | 1.11***  (1.05, 1.18) |  | 1.61 ***  (1.41, 1.84) | 1.00  (0.98, 1.01) |
| Physicians density at county hospitals | 0.99  (0.92, 1.06) | 0.91*  (0.88, 0.95) |  | 1.38***  (1.29, 1.48) | 1.08***  (1.02, 1.15) |  | 1.03 **  (1.00, 1.05) | 0.98 **  (0.97, 0.99) |
| Covariates |  |  |  |  |  |  |  |  |
| Rural | 0.93  (0.83, 1.03) | 0.67***  (0.63, 0.72) |  | 1.14***  (1.08, 1.24) | 1.10***  (1.04, 1.17) |  | 1.06**  (1.01, 1.11) | 1.04  (0.98, 1.09) |
| Male | 0.83***  (0.78, 0.88) | 0.94***  (0.91, 0.98) |  | 0.95***  (0.93, 0.98) | 0.99  (0.96, 1.03) |  | 0.82***  (0.79, 0.84) | 0.74***  (0.72, 0.77) |
| Age | 1.01***  (1.00, 1.01) | 1.02  (0.97, 1.07) |  | 1.00  (0.99, 1.00) | 0.99***  (0.98, 0.99) |  | 1.00***  (0.99, 1.00) | 0.99***  (0.99, 1.00) |
| Marriage status (Reference=single) |  |  |  |  |  |  |  |  |
| Married | 1.33***  (1.15, 1.55) | 0.97  (0.88, 1.08) |  | 1.69***  (1.58, 1.81) | 1.18***  (1.07, 1.31) |  | 2.06***  (1.91, 2.21) | 1.12  (0.97, 1.29) |
| Divorced or widowed | 1.17*  (1.00, 1.38) | 0.93  (0.84, 1.03) |  | 2.09***  (1.94, 2.26) | 1.27***  (1.14, 1.42) |  | 1.94***  (1.79, 2.10) | 1.51***  (1.30, 1.76) |
| Education (Reference=no formal education) |  |  |  |  |  |  |  |  |
| Primary school | 0.93  (0.86, 1.01) | 1.18***  (1.12, 1.24) |  | 0.99  (0.95, 1.03) | 0.94***  (0.90, 0.98) |  | 1.06***  (1.02, 1.11) | 1.09***  (1.05, 1.13) |
| Junior and high school | 0.68***  (0.54, 0.87) | 1.21***  (1.08, 1.37) |  | 0.74***  (0.69, 0.78) | 0.78***  (0.73, 0.83) |  | 0.95**  ()0.89, 1.00) | 0.94**  (0.89, 0.99) |
| Junior college and above | 0.49***  (0.35, 0.69) | 1.33***  (1.14, 1.54) |  | 0.52***  (0.45, 0.60) | 0.56***  (0.48, 0.65) |  | 0.83***  (0.74, 0.92) | 0.81***  (0.73, 0.91) |
| Employment status (Reference=farmers) |  |  |  |  |  |  |  |  |
| Unemployed | 1.09***  (1.02, 1.17) | 1.02  (0.97, 1.06) |  | 0.86***  (0.83, 0.90) | 0.89***  (0.85, 0.93) |  | 1.25***  (1.20, 1.29) | 0.99  (0.95, 1.03) |
| Informal employed | 0.76***  (0.66, 0.88) | 1.02  (0.95, 1.10) |  | 0.84***  (0.78, 0.90) | 0.85***  (0.79, 0.91) |  | 0.81***  (0.76, 0.86) | 0.83***  (0.78, 0.88) |
| Formal employed | 0.82***  (0.72, 0.93) | 0.94*  (0.87, 1.01) |  | 0.93**  (0.87, 0.99) | 0.91***  (0.85, 0.98) |  | 0.92***  (0.85, 0.97) | 0.99  (0.93, 1.04) |
| Household income per capita (Reference=the lowest level) |  |  |  |  |  |  |  |  |
| Lower-level | 1.05  (0.96, 1.18) | 1.05*  (0.99, 1.11) |  | 1.14***  (1.09, 1.19) | 1.14***  (1.09, 1.20) |  | 1.05**  (1.00, 1.09) | 1.02  (0.97, 1.07) |
| Middle-level | 0.97  (0.89, 1.07) | 1.08***  (1.02, 1.14) |  | 1.12***  (1.07, 1.17) | 1.10***  (1.04, 1.15) |  | 1.08***  (1.03, 1.12) | 1.02  (0.97, 1.06) |
| Higher-level | 0.82***  (0.74, 0.90) | 1.06*  (1.00 ,1.12) |  | 1.13***  (1.08, 1.19) | 1.08***  (1.02, 1.13) |  | 1.08***  (1.03, 1.12) | 1.06**  (1.01, 1.12) |
| The highest-level | 0.82***  (0.74, 0.92) | 1.00  (0.94, 1.07) |  | 1.09***  (1.03, 1.15) | 1.04  (0.98, 1.11) |  | 1.14***  (1.09, 1.20) | 1.06**  (1.01, 1.12) |
| Health insurance status (Reference=NCMS) |  |  |  |  |  |  |  |  |
| URRMS | 0.88**  (0.79, 0.99) | 1.05  (0.98, 1.11) |  | 1.06**  (1.01, 1.12) | 1.02**  (1.00, 1.05) |  | 1.06**  (1.01, 1.12) | 1.12***  (1.09, 1.15) |
| URBMI | 0.99  (0.86, 1.13) | 1.16***  (1.08, 1.26) |  | 0.93**  (0.87, 1.00) | 0.87**  (0.80, 0.95) |  | 1.00  (0.94, 1.06） | 0.98  (0.92, 1.05) |
| UEBMI | 1.07  (0.93, 1.24) | 1.08*  (1.00, 1.17) |  | 1.26***  (1.20, 1.33) | 1.27***  (1.21, 1.33) |  | 1.41***  (1.32, 1.50) | 1.39***  (1.30, 1.48) |
| Log (Distance to the nearest healthcare provider) | 1.07***  (1.04, 1.11) | 0.99  (0.98. 1.01) |  | 0.94***  (0.92, 0.95) | 1.00  (0.97, 1.04) |  | 1.00  (0.98, 1.01) | 1.00  (0.99, 1.02) |
| Presence of chronic disease (Reference=no) |  |  |  |  |  |  |  |  |
| Yes | 1.10***  (1.03, 1.16) | 1.20***  (1.15, 1.25) |  | 4.63***  (4.49, 4.78) | 4.39***  (4.25, 4.53) |  | 3.89***  (3.78, 4.01) | 3.91***  (3.80, 4.02) |

*p<0.05，**p<0.01，***p<0.001

**Appendix Table 5** Impact of health insurance and health workforce on choice of healthcare providers among outpatient care before and after China’s health system reform

| Outpatient choice of healthcare providers (reference group is village/community health stations) | Before China’s health system reform (RRR) | | |  | After China’s health system reform (RRR) | | |
| --- | --- | --- | --- | --- | --- | --- | --- |
|  | Township/  community health centers | County hospitals | Municipal or higher-level hospitals |  | Township/  community health centers | County hospitals | Municipal or higher-level hospitals |
| ARR at PHC institutions | 1.44  (0.96, 2.16) | 0.28***  (0.18, 0.44) | 0.18***  (0.10, 0.31) |  | 1.76 ***  (1.04, 2.74) | 1.07  (0.80, 1.44) | 0.98  (0.73, 1.32) |
| ARR at county hospitals | 1.31  (0.71, 2.39) | 1.68  (0.90, 3.15) | 2.92***  (1.51, 5.66) |  | 1.44*  (1.00, 2.08) | 1.72***  (1.20, 2.45) | 1.87***  (1.27, 2.74) |
| Physicians density at PHC institutions | 1.23***  (1.15, 1.32) | 0.43***  (0.30, 0.61) | 0.76  (0.43, 1.33) |  | 1.34  (0.80, 2.24) | 0.95***  (0.91, 0.99) | 0.98  (0.95, 1.02) |
| Physicians density at county hospitals | 1.02  (0.98, 1.06) | 1.38***  (1.29, 1.48) | 1.45 ***  (1.33, 1.58) |  | 0.91***  (0.89, 0.94) | 0.98  (0.95, 1.01) | 0.99  (0.96, 1.02) |
| Covariates |  |  |  |  |  |  |  |
| Rural | 0.99  (0.88, 1.12) | 0.74***  (0.65, 0.84) | 0.50***  (0.42, 0.59) |  | 1.02  (0.91, 1.26) | 0.76***  (0.67, 0.86) | 0.44***  (0.36, 0.53) |
| Male | 1.00  (0.94, 1.06) | 0.94  (0.88, 1.01) | 0.93  (0.84, 1.03) |  | 1.03  (0.97, 1.10) | 0.87***  (0.78, 0.97) | 0.96  (0.91, 1.04) |
| Age | 1.00  (1.00, 1.01) | 0..98***  (0.98, 0.99) | 0.99***  (0.98, 0.99) |  | 1.04***  (1.03, 1.05) | 1.01***  (1.00, 1.01) | 0.93***  (0.92, 0.93) |
| Marriage status (Reference=single) |  |  |  |  |  |  |  |
| Married | 0.81**  (0.68, 0.97) | 0.91  (0.75, 1.12) | 0.81  (0.61, 1.07) |  | 0.84**  (0.70, 1.00) | 0.75  (0.55, 1.01) | 0.94  (0.77, 1.15) |
| Divorced or widowed | 0.74***  (0.61, 0.89) | 0.65***  (0.52, 0.80) | 0.58***  (0.43, 0.79) |  | 0.77***  (0.63, 0.92) | 0.67***  (0.53, 0.82) | 0.52***  (0.37, 0.73) |
| Education (Reference=no formal education) |  |  |  |  |  |  |  |
| Primary school | 1.13***  (1.04, .1.23) | 1.17***  (1.06, 1.30) | 1.16*  (0.99, 1.37) |  | 1.17***  (1.08, 1.27) | 1.10  (0.93, 1.31) | 1.20  (1.09, 1.34) |
| Junior and high school | 1.12**  (1.02, 1.24) | 1.23***  (1.10, 1.39) | 1.31***  (1.09, 1.57) |  | 1.16***  (1.06, 1.28) | 1.27***  (1.13, 1.43) | 1.25***  (1.03, 1.51) |
| Junior college and above | 1.22  (0.79, 1.89) | 1.63**  (1.10, 2.42) | 1.60**  (1.04, 2.48) |  | 1.26  (0.82, 1.96) | 1.68***  (1.13, 2.49) | 1.54  (0.98, 2.41) |
| Employment status (Reference=farmers) |  |  |  |  |  |  |  |
| Unemployed | 1.04  (0.96, 1.14) | 1.25***  (1.13, 1.38) | 1.46***  (1.25, 1.71) |  | 1.08  (0.99, 1.18) | 1.29***  (1.16, 1.42) | 1.40***  (1.19, 1.64) |
| Informal employed | 1.05  (0.89. 1.24) | 1.33***  (1.12, 1.58) | 1.60***  (1.28, 2.01) |  | 1.06  (0.92, 1.28) | 1.37***  (1.15, 1.15) | 1.54***  (1.22, 1.94) |
| Formal employed | 1.12  (0.95, 1.32) | 1.22**  (1.03, 1.45) | 1.69***  (1.35, 2.10) |  | 1.08  (0.90, 1.28) | 1.25***  (1.06, 1.49) | 1.63***  (1.29, 2.03) |
| Household income per capita (Reference=the lowest level) |  |  |  |  |  |  |  |
| Lower-level | 0.93  (0.84, 1.03) | 1.09  (0.96, 1.24) | 0.96  (0.79, 1.17) |  | 0.86**  (0.77, 0.97) | 1.12*  (0.99, 1.28) | 0.90  (0.73, 1.11) |
| Middle-level | 1.06  (0.95, 1.17) | 1.34***  (1.18, 1.53) | 1.22**  (1.00, 1.48) |  | 1.10*  (0.99, 1.21) | 1.38***  (1.21, 1.57) | 1.16  (0.94, 1.42) |
| Higher-level | 1.06  (0.95, 1.19) | 1.40***  (1.22, 1.60) | 1.38***  (1.13, 1.68) |  | 0.99  (0.88, 1.13) | 1.34***  (1.15, 1.54) | 1.22  (0.97, 1.51) |
| The highest-level | 1.07  (0.95, 1.21) | 1.70***  (1.48, 1.97) | 1.91***  (1.56, 2.35) |  | 1.01  (0.89, 1.15) | 1.75***  (1.52, 2.03) | 1.84***  (1.50, 2.28) |
| Health insurance status (Reference=NCMS) |  |  |  |  |  |  |  |
| URRMS | 1.40***  (1.25, 1.57) | 1.31***  (1.14, 1.50) | 1.90***  (1.55, 2.32) |  | 1.25**  (1.09, 1.43) | 1.35***  (1.17, 1.54) | 1.84***  (1.49, 2.25) |
| URBMI | 1.09  (0.92, 1.30) | 1.61***  (1.35, 1.91) | 1.81***  (1.45, 2.27) |  | 1.13  (0.95, 1.35) | 1.66***  (1.39, 1.97) | 1.74***  (1.38, 2.20) |
| UEBMI | 1.89***  (1.58, 2.25) | 2.40***  (2.01, 2.87) | 2.39***  (1.89, 3.02) |  | 0.96  (0.63, 1.33) | 1.47**  (1.06, 1.95) | 2.32***  (1.82, 2.95) |
| Log (Distance to the nearest healthcare provider) | 1.19***  (1.15, 1.24) | 1.20***  (1.15, 1.25) | 1.09***  (1.02, 1.16) |  | 1.23***  (1.19, 1.28) | 1.23***  (1.18, 1.29) | 1.03  (0.96, 1.10) |
| Presence of chronic disease (Reference=no) |  |  |  |  |  |  |  |
| Yes | 1.41***  (1.32, 1.51) | 1.99***  (1.83, 2.16) | 1.74***  (1.54, 1.97) |  | 1.46***  (1.37, 1.56) | 2.05***  (1.88, 2.22) | 1.67***  (1.48, 1.90) |

*p<0.05，**p<0.01，***p<0.001

**Appendix Table 6** Impact of health insurance and health workforce on choice of healthcare providers among inpatient care before and after China’s health system reform

| Inpatient choice of healthcare providers (reference group is township/community health centers) | Before China’s health system reform (RRR) | | |  | After China’s health system reform (RRR) | | |
| --- | --- | --- | --- | --- | --- | --- | --- |
|  | County  hospitals | Municipal hospitals | Provincial or higher-level hospitals |  | County  hospitals | Municipal hospitals | Provincial or higher-level hospitals |
| ARR at PHC institutions | 0.75**  (0.57, 0.99) | 0.55***  (0.37, 0.82) | 0.54***  (0.36, 0.82) |  | 0.54***  (0.44, 0.66) | 0.19***  (0.15, 0.24) | 0.37***  (0.27, 0.50) |
| ARR at county hospitals | 1.22***  (1.06, 1.41) | 1.76***  (1.27, 2.45) | 1.85***  (1.34, 2.56) |  | 1.12  (0.91, 1.38) | 1.68***  (1.39, 2.03) | 2.18***  (1.74, 2.73) |
| Physicians density at PHC institutions | 0.90***  (0.84, 0.97) | 0.84***  (0.78, 0.91) | 0.92*  (0.85, 1.00) |  | 0.27***  (0.19, 0.36) | 0.18***  (0.10, 0.30) | 0.28***  (0.16, 0.48) |
| Physicians density at county hospitals | 1.41***  (1.29, 1.53) | 1.48***  (1.34, 1.64) | 1.52***  (1.37, 1.69) |  | 1.12***  (1.07, 1.17) | 1.14***  (1.09, 1.20) | 1.16***  (1.11, 1.22) |
| Covariates |  |  |  |  |  |  |  |
| Rural | 0.45***  (0.33, 0.62) | 0.42***  (0.29, 0.62) | 0.45***  (0.27, 0.76) |  | 0.44***  (0.32, 0.62) | 0.45***  (0.32, 0.65) | 0.45***  (0.26, 0.77) |
| Male | 1.00  (0.87, 1.16) | 1.13  (0.94, 1.39) | 1.01  (0.77, 1.34) |  | 1.01  (0.87, 1.18) | 1.26***  (1.07, 1.52) | 0.92  (0.67, 1.25) |
| Age | 0.98***  (0.98, 0.99) | 0.99***  (0.98, 0.99) | 0.97***  (0.96, 0.98) |  | 0.99**  (0.99, 1.00) | 1.14  (0.98, 1.36) | 0.87*  (0.77, 1.01) |
| Marriage status (Reference=single) |  |  |  |  |  |  |  |
| Married | 1.14  (0.76, 1.70) | 0.85  (0.50, 1.45) | 1.32  (0.62, 2.84) |  | 1.16  (0.76, 1.74) | 0.98  (0.64, 1.58) | 1.23  (0.51, 2.80) |
| Divorced or widowed | 0.95  (0.61, 1.47) | 0.63  (0.35, 1.14) | 0.71  (0.31, 1.66) |  | 0.96  (0.61, 1.50) | 0.76  (0.48, 1.27) | 0.61  (0.19, 1.58) |
| Education (Reference=no formal education) |  |  |  |  |  |  |  |
| Primary school | 1.04  (0.86, 1.26) | 1.49***  (1.11, 2.00) | 1.02  (0.67, 1.56) |  | 1.05  (0.86, 1.28) | 1.62***  (1.24, 2.13) | 0.93*  (0.89, 1.00) |
| Junior and high school | 1.17  (0.87, 1.59) | 1.55**  (1.02, 2.36) | 1.21  (0.68, 2.15) |  | 1.19  (0.88, 1.62) | 1.68***  (1.15, 2.49) | 1.12  (0.57, 2.09) |
| Junior college and above | 1.54  (0.52, 4.61) | 2.54  (2.78, 8.24) | 1.99  (0.52, 7.56) |  | 1.57  (0.51, 4.75) | 2.67***  (1.91, 4.34) | 1.92  (0.41, 3.66) |
| Employment status (Reference=farmers) |  |  |  |  |  |  |  |
| Unemployed | 1.15  (0.85. 1.55) | 1.39***  (1.08, 1.79) | 2.02***  (1.42, 2.87) |  | 1.17  (0.85, 1.58) | 1.52***  (1.21, 1.92) | 1.96***  (1.34, 2.83) |
| Informal employed | 1.14  (0.81, .159) | 1.24  (0.80, 1.91) | 0.85  (0.45, 1.61) |  | 1.16  (0.81, 1.62) | 1.37  (0.93, 2.04) | 0.75  (0.33, 1.53) |
| Formal employed | 1.31  (0.92, 1.88) | 1.63**  (1.06, 2.50) | 1.62*  (0.91, 2.89) |  | 1.33  (0.92, 1.92) | 1.76***  (1.19, 2.63) | 1.54  (0.81, 2.85) |
| Household income per capita (Reference=the lowest level) |  |  |  |  |  |  |  |
| Lower-level | 1.32**  (1.05, 1.66) | 1.01  (0.70, 1.46) | 1.05  (0.60, 1.82) |  | 1.34***  (1.06, 1.70) | 1.14  (0.83, 1.59) | 0.96  (0.49, 1.75) |
| Middle-level | 1.48***  (1.17, 1.86) | 2.02***  (1.43, 2.86) | 1.47  (0.86, 2.52) |  | 1.51***  (1.19, 1.90) | 1.49  (0.76, 2.47) | 2.15***  (1.56, 2.98) |
| Higher-level | 1.56***  (1.23, 1.98) | 1.74***  (1.22, 2.48) | 1.59*  (0.93, 2.72) |  | 1.59***  (1.25, 2.03) | 1.87***  (1.35, 2.61) | 1.51  (0.83, 2.68) |
| The highest-level | 1.65***  (1.27, 2.15) | 2.85***  (1.97, 4.13) | 3.53***  (2.07, 6.04) |  | 1.68***  (1.29, 2.20) | 2.98***  (2.10, 4.25) | 3.51***  (2.01, 6.09) |
| Health insurance status (Reference=NCMS) |  |  |  |  |  |  |  |
| URRMS | 1.71***  (0.58, 0.86) | 0.64***  (0.48, 0.85) | 1.03  (0.68, 1.56) |  | 0.77**  (0.62, 0.98) | 0.75***  (0.58, 0.87) | 0.94  (0.58, 1.48) |
| URBMI | 1.56***  (0.39, 0.79) | 0.47***  (0.30, 0.75) | 0.23***  (0.11, 0.48) |  | 0.59***  (0.38, 0.79) | 0.61***  (0.44, 0.88) | 0.21**  (0.09, 0.47) |
| UEBMI | 1.64**  (1.12, 2.39) | 1.37  (0.87, 2.16) | 1.52  (0.84, 2.75) |  | 1.67***  (1.14, 2.45) | 1.50*  (1.00, 2.29) | 1.44  (0.74, 2.71) |
| Log (Distance to the nearest healthcare provider) | 1.08*  (0.99, 1.17) | 0.97  (0.86, 1.11) | 1.06  (0.89, 1.27) |  | 1.09*  (1.00, 1.19) | 1.10*  (0.99, 1.24) | 0.97  (0.79, 1.18) |
| Presence of chronic disease (Reference=no) |  |  |  |  |  |  |  |
| Yes | 0.90  (0.77, 1.05) | 1.40***  (1.13, 1.74) | 1.57***  (0.65, 2.40) |  | 0.91  (0.77, 1.06) | 1.53***  (1.26, 1.87) | 1.49  (0.55, 2.35) |

*p<0.05，**p<0.01，***p<0.001
